# Molecular identification of clinical “difficult-to-identify” microbes from sequencing 16S ribosomal DNA and internal transcribed spacer 2

**DOI:** 10.1186/1476-0711-13-1

**Published:** 2014-01-03

**Authors:** Cancan Cheng, Jingjing Sun, Fen Zheng, Kuihai Wu, Yongyu Rui

**Affiliations:** 1 Laboratory Medicine Center, Nanfang Hospital, Southern Medical University, Guangzhou, Tonghe 510515, China

**Keywords:** Bacteria, Fungi, 16S rDNA, Internal transcribed spacer 2

## Abstract

**Background:**

Clinical microbiology laboratories have to accurately identify clinical microbes. However, some isolates are difficult to identify by the automated biochemical text platforms, which are called “difficult-to-identify” microbes in this study. Therefore, the ability of 16S ribosomal DNA (16S rDNA) and internal transcribed spacer 2 (ITS2) sequencing to identify these “difficult-to-identify” bacteria and fungi was assessed in this study.

**Methods:**

Samples obtained from a teaching hospital over the past three years were examined. The 16S rDNA of four standard strains, 18 clinical common isolates, and 47 “difficult-to-identify” clinical bacteria were amplified by PCR and sequenced. The ITS2 of eight standard strains and 31 “difficult-to-identify” clinical fungi were also amplified by PCR and sequenced. The sequences of 16S rDNA and ITS2 were compared to reference data available in GenBank by using the BLASTN program. These microbes were identified according to the percentage of similarity to reference sequences of strains in GenBank.

**Results:**

The results from molecular sequencing methods correlated well with automated microbiological identification systems for common clinical isolates. Sequencing results of the standard strains were consistent with their known phenotype. Overall, 47 “difficult-to-identify” clinical bacteria were identified as 35 genera or species by sequence analysis (with 10 of these identified isolates first reported in clinical specimens in China and two first identified in the international literature). 31 “difficult-to-identify” clinical fungi tested could be identified as 15 genera or species by sequence analysis (with two of these first reported in China).

**Conclusions:**

Our results show the importance of 16S rDNA and internal ITS2 sequencing for the molecular identification of “difficult-to-identify” bacteria and fungi. The development of this method with advantages of convenience, availability, and cost-effectiveness will make it worth extending into clinical practice in developing countries.

## Introduction

In clinical microbiology laboratories, traditional culture and biochemical techniques remain the primary methodology for identifying most pathogens. With the development of technology, automated microbiology system for the identification of pathogens has been introduced in many laboratories. In recent years, with the population aging, the number of patients with organ transplantation and bone marrow transplantation increasing, and immunosuppressive agents, anticancer drugs and invasive detection and treatment widely used, more and more patients suffer from severe infection of bacteria or fungi. Many clinical isolates from these patients can be identified by the automated biochemical text platforms in the most of laboratories. However, some isolates are difficult to identify by those platforms, which are called “difficult-to-identify” microbes in this study. Therefore, it is important to establish molecular diagnostic techniques for the identification of “difficult-to-identify” bacteria and fungi from clinical isolates.

Ribosomal genes encoding rRNA are present in the chromosomes of all prokaryotes and eukaryotes. In particular, 16S ribosomal DNA (rDNA)-based molecular identification has the potential to enable the identification of specific bacteria due to the universal distribution of 16S rDNA among bacteria and the presence of several special variable regions. The identification of organisms by sequencing DNA encoding 16S rRNA has been described in case reports that focus on the identification of different bacterial species or genera [1-4]. The internal transcribed spacer (ITS) region, ITS1-5.8-ITS2, located between the 18S and 28S rRNA genes and the 5.8S rRNA gene, have proved sufficient for the discrimination of most species of clinically important fungi. ITS regions, especially ITS2, have emerged as the most common target of molecular-based identification [5,6]. The development of an inexpensive and efficient application of molecular identification procedures would help with the clinical identification of “difficult-to-identify” bacteria and fungi according to 16S rRNA and ITS2 sequences.

Molecular identification methods, in which the partial sequence of 16S rDNA or ITS2 were sequenced and analyzed, were established in standard strains and clinical common isolates (including bacteria and fungi) in this study. In addition, isolates unidentified by routine laboratory procedures in our laboratory over the past 3 years were identified by these molecular methods.

## Methods

### Bacterial and fungal isolates

Bacterial and fungal isolates were obtained from the Laboratory Medicine Center of Nanfang hospital, a 2,200-bed university-affiliated tertiary-care institution. This laboratory routinely processes human samples for culture-based diagnosis of bacterial and fungal infectious diseases, including those due to anaerobes, aerobes, *Mycobacteria*, *Candida*, *Aspergillus*, and other isolates. All fungal isolates were isolated from deep mycosis. Conventional laboratory techniques for phenotyping were carried out on all isolates according to BD Phoenix 100 Automated Microbiology System (Becton, Dickinson and Co., Franklin Lakes, NJ) and MicroScan WalkAway® 96 *plus* System (Siemens Healthcare Diagnostics Inc., West Sacramento, CA). In this study, 78 “difficult-to-identify” microbes (including 47 bacteria and 31 fungi) from our laboratory were obtained from blood, puncture fluid, secretions, sputum and urine. All these isolates were difficult to identify by the automated biochemical text platforms in our laboratory during the past three years. *Staphylococcus aureus* ATCC29213, *Escherichia coli* ATCC25922, *Pseudomonas aeruginosa* ATCC27853, *Mycobacterium tuberculosis* H37Rv, *Candida albicans* ATCC90029, *Candida tropicalis* ATCC13803, *Candida glabrata* ATCC15126, *Candida parapsilosis* ATCC22019, *Aspergillus fumigatus* ATCC96918, *Aspergillus flavus* ATCC28539, *Aspergillus terreus* ATCC1012, *Aspergillus niger* ATCC16404, and other clinically common species were used as controls in this study. The study was approved by Medical Ethics Committee Nanfang Hospital Southern Med. Univ. and conducted in compliance with the Declaration of Helsinki.

### DNA extraction

Extraction of nucleic acids was carried out using a Lysis Buffer for Microorganism to Direct PCR kit (TaKaRa Bio Inc., Tokyo, Japan), supplemented by the TaKaRa MiniBEST Universal Genomic DNA Extraction Kit Ver.5.0 (TaKaRa), according to the manufacturer’s instructions. Colonies required collection from fresh culture in blood plates or Sabouraud’s media for bacteria and fungi, respectively. The concentration of colonies and hyphae in lysis buffer should also be within an appropriate range (visible turbidity). DNA previously extracted was directly amplified by PCR.

### 16S rDNA gene sequencing

The 16S rRNA genes were amplified from previous bacterial DNA using conventional PCR with primers 16S-F (5′-CCAGCAGCCGCGGTAATACG-3) and 16S-R (5′-ATCGG (C/T) TACCTTGTTACGACTTC-3′) [7], producing an amplicon of about 996 bp. PCR amplifications were performed using 5 L of template, 5 L of 10 × PCR buffer, 4 M of each primer stock solution, 4 mM of each dNTP, 1.25 U of ex Taq DNA polymerase (TaKaRa), and sterile distilled water added to a final total volume of 50 L. Amplification was performed using a Mastercycler® PCR System (Eppendorf International, Hamburg, Germany). Thermocycling parameters were 94°C for 10 min, 35 cycles of 94°C for 30 s, 55°C for 1 min, and 72°C for 2 min; a final extension step at 72°C was added for 5 min. The products of PCR amplification were examined by gel electrophoresis [100 V through a 1.5% agarose gel with 0.5 × TBE (Tris-borate-EDTA) running buffer], stained with ethidium bromide, and analyzed with the GelDoc XR Gel Documentation System (Bio-Rad, USA.). PCR amplicon sizes were estimated by comparison to molecular size markers (TaKaRa) then purified and sequenced (Sanger capillary sequencing) at the Beijing Genomics Institute (Shenzhen, China).

### ITS2 sequencing

The ITS2 regions were amplified from the previous fungi DNA using conventional PCR with primers ITS86-F (5′-GTGAATCATCGAATCTTTGAAC-3′) and ITS4-R (5′-TCCTCCGCTTATTGATATGC-3′) [8], producing an amplicon of approximately 200 ~ 400 bp. The PCR reaction mix, except for the primers, contained the same ingredients described above. Thermal cycling parameters were 10 min at 94°C, 30 cycles of 30 s at 94°C, 40 s at 55°C, and 1 min at 72°C; a final extension step at 72°C was added for 5 min. The products from PCR amplification were examined by gel electrophoresis as described above. Subsequent sequencing and analysis steps were performed as previously described.

### Sequence analysis

DNA sequences obtained were analyzed for homology using the BLASTN program at the National Center for Biotechnology Information website (http://blast.ncbi.nlm.nih.gov/Blast.cgi). Species and genera identification were presumed for clinical bacteria with a percent similarity of **≥**99% and **≥**97% [9], respectively, to reference sequences of strains in GenBank. Meanwhile, if % ID **≥** 99%, the highest E-value and % ID were taken as the criteria to identify the isolates. In addition, the strain was just identified as the level of genus when its result of the BLASTN showed more than two species in the same genus with % ID **≥**99%. A <97% identity indicated the isolate as a potentially new species.

The sequence of a fungus was assigned to a species if the best matching reference sequence showed **≥**98% homology and to a genus level on the basis of 95 to 98% homology to the best matching sequence or of **≥**98% homology with sequence entries for several species from the same genus. “No identification” was defined as <95% homology with the best matching reference sequence [10].

## Results

### Identification of bacteria by 16S rDNA sequence analysis

**Figure 1:**
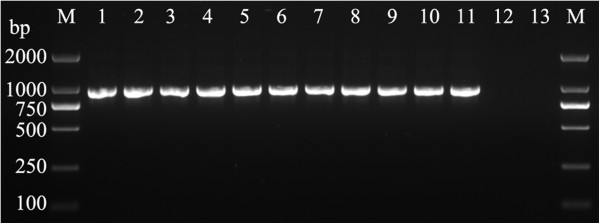
**Bacterial 16S rDNA PCR products of agarose gel electrophoresis.** The following DNA templates were used for PCR (by lane): 1, *Staphylococcus aureus* ATCC29213; 2, *Enterococcus faecalis*; 3, *Enterococcus faecium*; 4, *Streptococcus pneumoniae*; 5, *Streptococcus pyogenes*; 6, *Escherichia coli* ATCC25922; 7, *Klebsiella pneumoniae*; 8, *Pseudomonas aeruginosa* ATCC27853; 9, *Acinetobacter baumannii*; 10, *Bacteroides fragilis*; 11, *Mycobacterium tuberculosis* H37Rv; 12, *Candida albicans* ATCC 90029; 13, human leucocyte.

The 16 s rRNA amplicons of some isolates including 5 standard strains and clinical isolates showed on electropherograms (Figure 1). Sequencing results of the standard strains were consistent with the known phenotype. The clinically common isolates used in the study were *Staphylococcus epidermidis*, *Staphylococcus haemolyticus*, *Enterococcus faecium*, *Enterococcus faecalis*, *Streptococcus agalactiae*, *Streptococcus pneumonia*, *Streptococcus pyogenes*, *Klebsiella pneumoniae*, *Enterobacter cloacae*, *Enterobacter aerogenes*, *Proteus mirabilis*, *Serratia marcescens*, *Acinetobacter baumannii*, *Pseudomonas aeruginosa*, *Pseudomonas maltophilia*, *Burkholderia cepacia*, *Aeromonas hydrophila* and *Flavobacterium indologenes*. Sequencing results of these isolate were consistent with the phenotypic identification by the automated microbiology system.

A total of 47 “difficult-to-identify” bacterial isolates, including 22 Gram-negative bacteria, 23 Gram-positive and two acid-fast bacilli were analyzed by 16S rDNA PCR and sequencing. Sequencing identified 30 isolates as 25 species and the remaining 17 isolates as ten genera (Table 1). In these results, 10 isolates were first reported from clinical specimens in China and two were first reported in the international literatures.

**Table 1:** 16S rDNA-based identification of a collection of 47 “difficult-to-identify” bacteria clinical isolates

**Genus/strain**	**No. of isolates**	**Source of isolates**	**GenBank accession no.**	**BLAST**	
				**E-value**	**% ID**
Gram-negative	22				
*Acinetobacter johnsonii*	1	Secretion	KF381212	0.0	100
*Aggregatibacter aphrophilus*	1	Blood	KF410885	0.0	100
*Agrobacterium tumefaciens*^a^	1	Blood	JF487939	0.0	100
*Bacteroides fragilis*	1	Blood	KF381209	0.0	100
*Bilophila wadsworthia*^a,^	1	Pleural fluid	KF414628	0.0	99
*Campylobacter fetus*	1	Blood	KF372434	0.0	100
*Capnocytophaga sputigena*^a^	3	Blood, secretion	JF422019	0.0	99
*Delftia sp.*	1	Puncture fluid	KF410887	0.0	--^c^
*Dialister pneumosintes*^a^	1	Cerebrospinal fluid	KF410890	0.0	100
*Eikenella corrodens*	1	Blood	KF372435	0.0	100
*Enterobacter* sp*.*	1	Blood	KC148534	0.0	--^c^
*Haemophilus influenzae*	1	Blood	JF487936	0.0	100
*Massilia* sp.^a^	1	Puncture fluid	KF410886	0.0	100
*Moraxella osloensis*	3	Blood, secretion	KF372432	0.0	100
*Neisseria meningitidis*	2	Cerebrospinal fluid, blood	KF372431	0.0	99
*Pontibacter* sp.^b^	1	Puncture fluid	KF410889	0.0	--^c^
*Prevotella bivia*	1	Secretion	KF372433	0.0	99
Gram-positive	23				
*Bacillus amyloliquefaciens*	1	Puncture fluid	KF410881	0.0	99
*Bacillus niabensis*^b^	1	Puncture fluid	KF381217	0.0	100
*Bacillus pumilus*	1	Secretion	KF381216	0.0	99
*Brachybacterium* sp.	1	Secretion	KF410892	0.0	--^c^
*Corynebacterium amycolatum*	1	Blood	JF487935	0.0	99
*Corynebacterium falsenii*	1	Blood	KF414626	0.0	100
*Corynebacterium sp.*	2	Blood, secretion	KF414627	0.0	--^c^
*Corynebacterium xerosis*	1	Blood	KF410882	0.0	100
*Granulicatella adiacens*	1	Blood	KF381213	0.0	100
*Janibacter* sp.^a^	1	Blood	KF410884	0.0	--^c^
*Leifsonia* sp.^a^	2	Blood	KF410883	0.0	--^c^
*Nocardia* sp.	6	Secretion	KF381210	0.0	--^c^
*Paenibacillus massiliensis*^a^	1	Puncture fluid	KF381215	0.0	100
*Propionibacterium acnes*	1	Blood	KF381211	0.0	100
*Rhodococcus equi*	1	Secretion	KF410888	0.0	100
*Streptomyces* sp.	1	Secretion	KF410891	0.0	--^c^
Acid-fast staining positive^d^	2				
*Mycobacterium abscessus*	1	Secretion	JF487938	0.0	100
*Mycobacterium tuberculosis*	1	Sputum	KF410893	0.0	100

^a^The clinical isolates first reported in China.

^b^The clinical isolates first reported in the international literature.

^c^More than two species in the same genus with the % ID ≥ 99%.

^d^All these isolates are not available to be identified by Phoneix or Microscan, and exception of these were attempted to identify by Phoneix.

### Identification of clinical isolates by ITS2 sequence analysis

**Figure 2:**
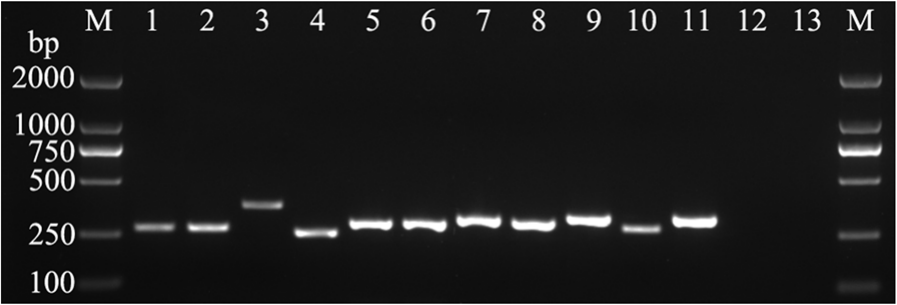
**Fungal ITS2 PCR products of agarose gel electrophoresis.** The following DNA templates were used for PCR (by lane): 1, *Candida albicans* ATCC90029; 2, *Candida tropicalis* ATCC13803; 3, *Candida glabrata* ATCC15126; 4, *Candida parapsilosis* ATCC22019; 5, *Aspergillus fumigatus* ATCC96918; 6, *Aspergillus flavus* ATCC28539; 7, *Aspergillus terreus* ATCC1012; 8, *Aspergillus niger* ATCC16404; 9, *Cryptococcus neoformans*; 10, *Fusarium* sp.; 11, *penicilliposis marneffei*; 12, *Escherichia coli* ATCC25922; 13, human leucocyte.

Electropherograms in Figure 2 shows the result of PCR amplicons from the ITS2 region including four eight standard strains and 3 “difficult-to-identify” isolates. The identification of these standard strains by sequencing was consistent with their phenotype.

A total of 31 “difficult-to-identify” fungal isolates from various clinical specimens were analyzed by ITS2 PCR and sequencing. Sequencing identified these isolates as 15 different genera or species (Table 2). Of these clinical fungi isolates, 27 isolates were identified as 13 species and 5 were identified as two genera. Two isolates were first reported from clinical specimens in China.

**Table 2:** ITS2-based identification of a collection of 31 fungi “difficult-to-identify” clinical isolates

**Genus/strain**^**d**^	**No. of isolates**	**Source of isolates**	**GenBank accession no.**	**BLAST**	
				**E-value**	**% ID**
*Aspergillus flavus*	1	Secretion	KF425318	7e-131	100
*Aspergillus fumigatus*	2	Sputum	JF487933	9e-130	100
*Aspergillus niger*	1	Sputum	KF425317	2e-132	100
*Aspergillus terreus*	2	Sputum, secretion	KF425307	1e-133	99
*Cryptococcus neoformans*	1	Blood	KF425316	3e-140	100
*Exophiala jeanselmei*	1	Sputum	KF425314	5e-138	100
*Fusarium* sp*.*	3	Blood, secretion	JF487937	2e-136	--^c^
*Penicillium marneffei*	9	Blood, sputum, secretion	JF422020	5e-148	100
*Phomopsis* sp.^a^	1	Secretion	KF425311	1e-122	--^c^
*Rhizopus oryzae*	1	Secretion	KF425308	4e-139	100
*Rhodotorula mucilaginosa*	1	Blood	KF425309	1e-158	100
*Trichomonascus ciferrii*^a^	1	Secretion	KF425312	3e-135	100
*Trichosporon asahii*	5	Blood, urine, secretion	KF425306	7e-126	100
*Trichosporon asteroides*	1	Blood	KF425313	6e-132	100
*Trichosporon jirovecii*	1	Secretion	KF425310	9e-130	100

^a^The clinical isolates first reported in China.

^c^More than two species in the same genus with the % ID ≥ 98%.

^d^All these isolates are not available to be identified by Phoneix or Microscan.

## Discussion

Most isolates recovered in our laboratory are identified by phenotypic criteria with an automated microbiology system, which likely reflects the present situation at most teaching hospital laboratories worldwide. It is well known that the microbiology systems from Siemens MicroScan WalkAway 96 *plus* System, BD Phoenix 100 Automated Microbiology System and BioMerieux Vitek 2 Compact have been extensively applied clinically in different regions. However, some isolates that are uncommon or atypical clinically have difficulty in identifying by the above systems. Genetic methods for the identification and taxonomic classification of microorganisms have thus been established in clinical microbiology laboratories in recent years.

The sequence analysis of 16S rDNA is the most common molecular method for the identification of bacteria [11], but most studies have mainly focused on method development and anecdotal case reports. On the basis of key reports and previous clinical work, we selected a set of universal reference primers to amplify 16S rDNA and identify previously “difficult-to-identify” bacteria in clinical settings. In addition, the ITS region (especially ITS2) is heavily used for the identification of fungi [12]. The identification of standard strains and common clinical isolates by PCR and sequencing in this study demonstrates that this method is consistent with conventional laboratory methods. This molecular identification method offers numerous desirable features: (1) convenience, as common molecular biology methods and techniques are used in the procedure (including efficient one-step DNA extraction, PCR, electrophoresis, commercial sequencing, and sequence analysis); (2) Availability, as it is based on well established theories and methods (including amplicon preparation by PCR, commercial sequencing, and GeneBank verification); and (3) cost-effectiveness, as the cost of the procedure can be lower than ever for the development of information and technology. If laboratories haven’t the extensive expertise to use other methods by phenotypic criteria, the use of the molecular methods as an alternative to identify “difficult to identify” microbes can be considered.

Various bacteria were identified through sequencing in this study. The overall performance of sequence analysis is excellent, as it enables the identification of “difficult-to-identify” bacterial isolates. Thus, the sequencing of 16S rDNA can serve as a supplement in regular clinical work for bacteria identification, especially for poorly described, biochemically deficient or fastidious organisms. However, the method does have a little potential limitation that it can not identify all of bacteria successfully. This is due to insufficient base heterogeneity in the 16S rDNA of some organisms, resulting in the inadequate discrimination of some species, such as *Shigella sonnei* and *Escherichia fergusonii*[13]. In this case, epidemiological, clinical, and phenotypic microbiological data of an isolate can be collected and combined with sequence analysis from a more variable region, such as intergenic 16S-23S rRNA spacer regions.

The conventional identification of fungi is mainly based on a combination of morphological and biochemical features. Morphologies at all levels require careful expert evaluation for correct species assignment because of excessive morphological variability. Our study demonstrates the feasibility of ITS2 sequencing for the identification of clinically important and rare fungi. This method identified 31 isolates as 15 species or genera, including *Aspergillus*, *Cryptococcus*, *Exophiala*, *Rhizopus*, and *Trichosporon*. Almost all clinically relevant species could be identified by ITS2 region sequencing, which is highly specific [5].

We conducted a systematic search in PubMed, GenBank, and Chinese National Knowledge Infrastructure (CNKI) databases to review case reports related to rare clinical isolates we identified. One of these rare isolates, from a female patient with acute pancreatitis, is *Agrobacterium tumefaciens*, a rod shaped, Gram-negative soil bacterium and the causal agent of crown gall disease. Although the international literature has reported *Agrobacterium tumefaciens* in clinical catheter-related infection [14], this clinical infection has not been reported in China. Another rare isolate, from puncture fluid of a lung cancer patient with pleural effusion, *Bilophila wadsworthia*, is a unique Gram-negative anaerobic rod bacterium that has not been reported in mainland China but in Taiwan [15]. International literature has reported this isolate from a variety of specimens like bile, pus, and blood [16,17]. *Capnocytophaga sputigena* is a Gram-negative rod bacterium in the oral flora of healthy humans that likely causes systemic infections in immunocompromised patients with granulocytopenia and oral ulcerations, especially in children [18,19]. In this study, three isolates were recovered at our hospital from three children (under 10 years old) suffering from lymphoblastic leukemia or thalassemia. *Dialister pneumosintes*, a small, non-fermentative, Gram-negative anaerobic rod bacterium, is considered a commensal organism of the oral cavity, nasopharynx, intestine, and vaginal flora [20,21]. This pathogen was responsible for various infections in immunocompromised patients [22]. A 50-year-old woman suffering from cerebral trauma was infected by *D. pneumosintes* isolated from cerebrospinal fluid in this study. A species of *Massilia*, *Massilia timonae*, a nonfermentative aerobic Gram-negative rod bacterium, was isolated from the blood and CSF [23,24]. We first reported *Massilia* sp*.* from clinical specimens in China.

*Pontibacter* sp*.* has been mainly reported in the environment, including water, garbage dumps, and soil. There are no clinical reports on this species in the international literature to date. *Bacillus niabensis* is also a species commonly found in the environment that rarely causes clinical infections and human colonization. Here, the specimens from bathystixis contained *B. niabensis* in a patient. The genus *Janibacter* is typically isolated from sludge and the name *Janibacter* refers to the changing morphology of the microorganisms during growth [25,26]. We identified *Janibacter* sp*.* from an adult with a fever over 38°C, indicating it may be isolated from clinical specimens, even blood, in China. *Leifsonia* sp*.* is an aquatic coryneform rod rarely associated with human infections. Some species, like *Leifsonia aquatica*, have been accidentally found in the clinic in other countries, but no species previously reported in China [27-29]. We report two cases of bloodstream infection in a female patient with osteogenic sarcoma and a 45-day-old infant. *Paenibacillus massiliensis* is a Gram-positive, facultative anaerobic rod bacterium isolated from blood culture [30]. We are the first to report the species from clinical specimens in China. In this hospital, the sample containing acid-fast bacilli will be sent to Guangzhou Antituberculosis Station to identify the microbes after our laboratory checks it positive by acid-fast staining. This *Mycobacterium tuberculosis* in Table 1 was used to validate the molecular method of 16S rDNA sequencing.

*Phomopsis* sp*.* is a frequent fungal parasite of plants around the world, but only a limited number of genera have been documented to cause human disease [31-33]. We report the first human case due to *Phomopsis* sp*.* in China. *Rhizopus oryzae* can cause invasive fungal infections called mucormycosis in humans [34]. Here we report a serious case of disseminated type mucormycosis caused by *Rhizopus oryzae* in a patient suffering diabetes with orbital cellulitis, which is a rare clinical case. *Trichomonascus ciferrii* known as *Candida ciferrii* has morphological characteristics that differ from more common *Candida* species and is emerging as a causative agent of opportunistic infections. This is the first case reported in China, from a patient with chronic suppurative otitis media.

In this study, all “difficult-to-identify” isolates were correctly identified and the etiological agent identified. Therefore, sequencing 16S rDNA and ITS 2 region is a good supplemental for clinical conventional methods in the identification of microbes.

## Consent

Written informed consent was obtained from the patient for the publication of this report and any accompanying images.

## Competing interests

The authors declare that they have no competing interests.

## Authors’ contributions

CC and JS carried out the evaluation of experiments, data organization and analysis and contributed to writing and to the interpretation of the results. FZ collected all of bacteria isolates and clinical data; KW collected all of fungi isolates and clinical data. YR contributed to the design of the study and assisted in the drafting of the manuscript. All authors have read and approved the manuscript.
